# Predictors of back disorder among Almeda textile factory workers, North Ethiopia

**DOI:** 10.1186/s13104-018-3440-4

**Published:** 2018-05-16

**Authors:** Teklehaymanot Huluf Abraha, Asmelash Tekie Demoz, Haimanot Gebrehiwot Moges, Ansha Nega Ahmmed

**Affiliations:** 1 School of Public Health, College of Health Sciences, Aksum University, P.o.Box: 1010, Aksum, Ethiopia; 2 Adwa General Hospital, Adwa, Ethiopia; 3 Department of Environmental and Occupational Health and Safety, College of Medicine and Health Sciences, University of Gondar, Gondar, Ethiopia

**Keywords:** Textile factory, Back disorder, Musculoskeletal disorders, Ethiopia

## Abstract

**Objectives:**

To guide the development of targeted interventions for the prevention of work-related back pain, this manuscript estimates the prevalence of back pain and its association with a variety of risk factors among Almeda textile factory production works from March to April 2015. An institutional—based cross-sectional study was carried out in Almeda textile factory, North Ethiopia. Randomly selected workers were administered a structured questionnaire about their socio-economic status, lifestyle, working conditions, back pain and selected risk factors. The data was entered to Epi Info 3.5.4 version and analyzed using SPSS version 16. Descriptive statistics were done to characterize the study participants. Bivariate and multiple logistic regressions were fitted to control confounding variables. Adjusted odds ratio with 95% confidence intervals was computed.

**Results:**

The prevalence of work-related musculoskeletal disorders was 53.1%. Gender, age, years of service, lack of physical activity, unavailability of adjustable chair, work-load and poor light were significantly associated with increased risk of back pain. The high prevalence of work-related back pain disorder implies that; habit of doing physical exercise, availing adjustable chair and light at the working place, are key issues which require specific interventions.

**Electronic supplementary material:**

The online version of this article (10.1186/s13104-018-3440-4) contains supplementary material, which is available to authorized users.

## Introduction

Musculoskeletal disorders (MSDs) are injuries or pains in the body’s joints, ligaments, muscles, tendons, peripheral nerves, and supporting blood vessels [1, 2]. It is responsible for a large portion of worker’s compensation costs and is a primary source of lost production [3]. World wide, 37% of lower back pain (LBP) is attributable to occupational risk factors [4].

In developing countries where there is poor awareness of ergonomics issues, education, training program and certification makes it to under-report and accelerated the problem. Even though the textile industry provides job opportunity to a considerable section of the population, it also exposes workers to the occupational risk for back [5]. In the textile setting, where the workers perform task in prolonged standing, highly repetitive work, heavy lifting, working with the hands lifted to shoulder height or higher, and working with the back twisted or bent forward are the contributing factors to develop impaired work ability, musculoskeletal disorders and enhance long term sickness absence [6]. Research has shown gender [7–10], age [9–11], years of services [9] and adjustable chair [11], to be the key factors associated with back disorder.

In Ethiopia, industrial areas are increasing from time to time especially the textile industry. However, its status and factors affecting of occupational back pain have not been well studied in the study area. This study, therefore, was designed to assess the magnitude and associated factors of back pain which can provide potential practical guidance to prevent these health problems.

## Main text

### Methods

**Fig. 1:**
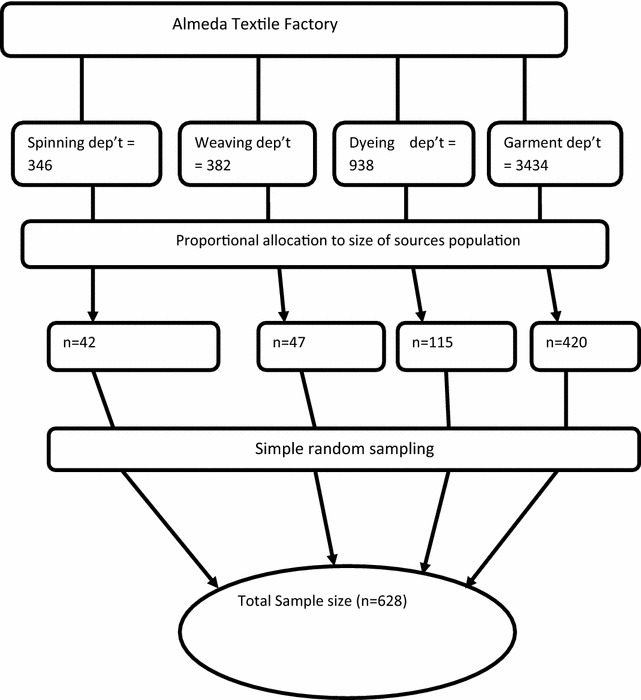
Schematic representation of sampling procedure, predictors of back disorder among Almeda textile factory workers, North Ethiopia

An institutional-based cross-sectional study design was conducted from March to April, 2015 among Almeda textile factory production workers. Almeda textile factory is situated 7 km from the center of Adwa town on the main road to Aksum and 1006 km from Addis Ababa the capital city of Ethiopia and 233 km from Mekelle, capital city of the Tigray regional state. The factory is established on February 1996. It is one of the biggest textile manufacturing companies in the country consisting of the spinning, weaving, dyeing and garment departments. It has a total of 5100 workers with 3600 (70%) females and 1500 (30%) males. All production workers who had worked in the textile factory for more than 12 months prior to the study were taken as source population. The sample size was determined using a single population proportion formula [12], 50% for back pain with 95% confidence interval and margin of error 4% between the sample and the underlying population, 5% none response. Computing with the above formula gives a total sample size of 624. After production workers were stratified by their department, the total sample was proportionally allocated to each department according to their size (Fig. 1). Each study participant was selected randomly by using computer generated random number. A structured questionnaire derived from Standard Nordic Questionnaire and literature reviews (Additional file 1) was used for data collection as a tool for gathering data on the occurrence of musculoskeletal symptoms [13].

Three data collectors (BSc Nurse Holders) and one resident supervisor participated to collect the data. Before the actual data collection, pretest was conducted with 5% of the study participants in Mekelle City Administrative (My-Garment Textile PLC) and necessary corrections were made.

The data was entered to Epi Info 3.5.4 and analyzed using SPSS version 16. For the descriptive analysis, continuous variables were summarized using means and standard deviations, while categorical variables were summarized using proportions. Bivariate and multiple logistic regressions were used to determine the effect of independent variables on the outcome variable of association using adjusted odds ratio (AOR) with 95% confidence interval (CI). P-value  < 0.05 considered as statistical significant. The assumption of binary logistic regression model was conducted. Multicollinearity was checked using the Variance Inflation Factors (VIF) and it is small (2.1) which indicated the absences of collinearity among predictors. In addition, the goodness of model fitness was tested using Hosmer–Lemeshow test which was yield a large P-value (0.52) and the result of the regression analysis was suggested an evidence for model adequacy well fitted with the predictors.

### Operational terms

#### Back pain musculoskeletal disorders

Self-reported musculoskeletal symptoms on lower and/or upper back are defined by aches, pain, or discomfort during the past 12 months preceding completion of the questionnaire.

#### Repetitive work

Worker repeats the same motion with less than 30 s with little or no variation for more than 2 h total per day.

### Results

#### Socio-demographic characteristics of production workers

Six hundred eighteen of the study participants were responded to the questionnaire making the response rate 99%. Most of the workers 420 (68%) were involved in the garment department. Majority of the participants, 433 (70.1%) were females and 432 (64.4%) were in the age group between 25 and 33 years. Three hundred forty-nine (56.5%) of the workers were married and 232 (37.5%) single. The majority of (44.8%) of the study participants were attended secondary school and one-third of (33.5%) were attended Technical and Vocational Education Training (TVET). Two hundred eighty (45.3%) and 38 (6.1%) of the employees were served from 6 to 10 years and above 15 years respectively (Table 1).

**Table 1:** Socio-demographic characteristics of Almeda textile factory production workers, North of Ethiopia, June, 2015 (n = 618)

Category of variable	Frequency (n = 618)	Percent (%)
Gender		
Male	185	29.9
Female	433	70.1
Age		
Mean (SD)	29.99 (± 4.822)	
> 25 years	56	9.1
25–33 years	398	64.4
34–42 years	149	24.1
≥ 43 years	15	2.4
Marital status		
Married	349	56.5
Single	232	37.5
Others^a^	37	6.0
Educational level		
Primary	91	14.7
Secondary	277	44.8
Technique	207	33.5
Higher education	43	7.0
Monthly salary (USD)		
< 37	146	23.6
37–74	453	73.3
> 74	19	3.1
Year of service in textile		
Mean (± SD)	7.19 (± 4.173)	
1–5 years	180	29.1
6–10 years	280	45.3
11–15 years	120	19.4
> 15 years	38	6.1
Department		
Spinning and weaving	88	14.2
Garment	420	68.0
Dyeing	110	17.8

*SD* standard deviation, *USD* United States Dollar

^a^Divorced, widowed, separated

#### Personal characteristics of workers

Majority of the respondents, 446 (72.2%) were being healthy (18.5–24.9 kg/m^2^) and 164 (26.5%) under-weight (> 18.5 kg/m^2^). Five hundred thirty-one (85.9%) of the workers were not practicing physical exercise, 36 (5.8%) practice three and above times per week. Most of the workers, 590 (95.5%) were non-cigarette smokers and only 12 (1.9%) smoke cigarette 1–3 days per week.

#### Ergonomic and working conditions of workers

Sixty-five (10.5%) and more than one-third (36.9%) of the workers had always exposed to repetitive task and work load respectively. One hundred eighty-seven (30.3%) of the respondents were not satisfied with their current job, 198 (30.9%) had poor light to operate, and 171 (27.7%) of the workers were not using an adjustable chair.

#### Organizational factors of production workers

One hundred fifty two (24.6%) of the workers work for nine and above hours a day. 428 (69.3%) of the workers didn’t have work break, 34 (5.5%) had below 15 min per shift/day.

#### Prevalence of work related back disorder among production workers

The prevalence of work-related back musculoskeletal disorders among production workers who had experienced trouble (ache, pain and discomfort) in the last 12-months period was 53.1% (n = 328).

#### Associated factors for back MSDs

Female workers were almost 13 times more likely to develop back disorder as compared to males (AOR = 12.88; 95% CI 4.15, 40.0). The probability of developing back disorder were 2.89 times greater in workers with age of 25–33 years than workers with age < 25 years (AOR = 2.89; 95% CI 1.10, 7.98). Workers with service of 11–15 years were above 5 times more likely to develop back disorder than had short year of service (1–5 years) (AOR = 5.1; 95% CI 1.62, 16.13). Respondents who hadn’t the habit of doing physical activities were 10.94 times more likely to develop back MSD than had doing physical activities greater than three times a week (AOR = 10.94; 95% CI 1.85, 64.88).

Workers those who have no adjustable chair were 4.58 times more likely to develop back disorders than those with adjustable chair (AOR = 4.58; 95% CI 2.41, 8.75). Workers who had work load always developed back disorder more than 7 times compared to those who did not have (AOR = 7.45; 95% CI 2.92, 18.98) and those had work load some times were 2.62 times more likely to develop (AOR = 2.62; 95% CI 1.16, 5.91). Employees who perform their job in poor light were 2.54 times more likely to develop back disorder than who perform in enough light which enable to see fine details (AOR = 2.54; 95% CI 1.36, 4.73) (Table 2).

**Table 2:** Association of factors with work-related back MSD among Almeda textile factory production workers, June, 2015

Category of variables	Back disorder			
	Yes, n (%)	No, n (%)	COR (95% CI)	AOR (95% CI)
Gender				
Male	74 (40)	111 (60)	1.00	1.00
Female	254 (58.7)	179 (41.3)	2.13 (1.49–3.02)	12.88 (4.15–40.0)***
Age (years)				
< 25	14 (25)	42 (75)	1.00	1.00
25–33	213 (53.5)	185 (46.5)	3.45 (1.83–6.53)	2.89 (1.10–7.98)*
34–42	90 (60.4)	59 (39.6)	4.60 (2.29–9.1)	1.34 (0.36–4.91)
≥ 43	11 (73.3)	4 (27.7)	8.20 (2.26–30.1)	0.67 (0.10–4.92)
Department				
Spinning and weaving	52 (59.1)	36 (40.9)	1.80 (1.02–3.17)	
Garment	227 (54)	193 (46)	1.46 (0.96–2.23)	
Dyeing	49 (44.5)	61 (55.5)	1.00	
Year of service in textile (years)				
1–5	56 (31.1)	124 (68.9)	1.00	1.00
6–10	161 (57.5)	119 (42.5)	2.99 (2.01–4.44)	2.08 (0.97–4.50)
11–15	85 (70.8)	35 (29.2)	5.38 (3.24–8.9)	5.1 (1.62–16.13)**
≥ 16	26 (68.4)	12 (31.6)	4.8 (2.23–10.19)	3.99 (0.84–18.93)
Educational status				
Primary school	67 (73.6)	24 (26.4)	4.27 (1.98–9.21)	
Secondary school	159 (57.4)	118 (42.6)	2.06 (1.07–3.97)	
Technique and vocational	85 (41)	122 (59)	1.07 (0.55–2.08)	
Higher education	17 (39.5)	26 (60.5)	1.00	
Physical activity				
None	310 (58.4)	221 (41.6)	8.69 (3.32–22.72)	10.94 (1.85–64.88)*
Once a week	13 (25.5)	38 (74.5)	2.12 (0.68–6.6)	3.14 (0.41–24.28)
≥ 3 times a week	5 (13.8)	31 (86.2)	1.00	1.00
BMI				
Underweight (< 18.5 kg/m^2^)	101 (61.6)	63 (38.4)	1.6 (1.11–2.31)	
Healthy (18.5–24.9 kg/m^2^)	227 (50)	227 (50)	1.00	
Availability of adjustable chair				
Yes	56 (32.75)	115 (67.25)	1.00	1.00
No	272 (60.85)	175 (39.15)	3.19 (2.2–4.63)	4.58 (2.41–8.75)***
Work load				
Never	19 (20.44)	74 (79.56)	1.00	1.00
Sometimes	124 (41.75)	173 (58.25)	2.79 (0.04–0.98)	2.62 (1.16–5.91)*
Always	185 (81.14)	43 (18.86)	16.75 (9.16–30.6)	7.45 (2.92–18.98)***
Availability of enough light				
Low	155 (78.28)	43 (21.72)	4.49 (2.88–6.99)	2.54 (1.36–4.73)**
High	85 (44.5)	106 (55.5)	1.00	1.00

*AOR, COR* adjusted and crude odd ratio respectively, *1.00* reference, *BMI* Body Mass Index

* P-value < 0.05, ** P-value < 0.01, *** P-value < 0.001

### Discussion

The prevalence of self-reported back disorder was 53.1% (95% CI 49, 58). This finding was consistent with a study done in Sri Lanka in which the prevalence of back was 57.3% [11]. However, this study finding was inconsistent with a study done in Bangladesh 68%. This disagreement could be that, the practice of occupational health and safety in Ethiopia is at its infancy stage; work-related disorders are under-diagnosed and under-reported. Therefore, participants may undermine their self reported MSDs. Female workers were more likely to develop back disorder (AOR = 12.88; 95% CI 4.15, 40.00) than males. This finding is in line with studies conducted in Bangladesh, Thailand, Los Angeles and Nepal [7–10]. The reason that females developed pain more than males could be that most of them are operators of sewing machines which demand prolonged sitting and beyond this they have to work too much at home while carrying their family which is double burden and they have no enough rest time to repair.

Employees with age of 25–33 years had more probability of developing back disorder than workers with age of less than 25 years (AOR = 2.89; 95% CI 1.10, 7.98). This reveals the fact that musculoskeletal disorders develop gradually through prolonged exposure (cumulative trauma). This result is supported by studies conducted in Iran and Nepal [10, 11]. A study done in Thailand revealed that younger workers develop back disorder more likely than older age which contradicts with this study finding [11, 14]. This could be due to the difference in study settings where the study in Thailand is conducted among office workers that incorporate varieties of tasks that don’t involve sitting or standing for prolonged hours. Employees with longer year of service (11–15 years) in the textile were more than 5 times more likely to develop back disorder than employees had short (1–5 years) year of service (AOR = 5.10; 95% CI 1.62, 16.13). This shows working for long years was strongly associated and increased work related back musculoskeletal disorders because of cumulative exposure. This result is consistent with a study conducted in Los Angeles [9].

Habit of doing physical activities were found significantly associated with work related back disorder. Employees those who had no habit of doing physical activities were 10.94 times more likely to develop work-related back disorder as compared to those who have had habit of doing physical activities for more than three times per week (Table 2). This indicates practicing physical activity makes muscles strong to resist spasm, stimulates blood vessels to run proper blood circulation that reduce vessel compression and help to overcome pain.

Participants who have work load always in the work place develop back disorder higher than who do not have work load. This reveals working with high demand of work was strongly associated and increased work-related back and shoulder musculoskeletal disorders. This is because, when there is work load, workers are prone to high pace of work, time pressure and lack of control over the tasks performed and hence, the chance of developing work-related MSD likely increases among those workers. This is in line with the studies conducted in Iran and USA, Los Angeles [9]. Workers those who had no adjustable chair were 4.58 times more likely to develop back disorder than as compare to those who have adjustable chair. This finding agrees with the study conducted in Iran among hand-woven carpet industry workers which reveals that MSDs in back and shoulders body regions were significantly associated with seat type or adjustable chair [11].

Employees who perform their job in poor light were more than twice more at risk to develop back MSD relative to workers performing their job in sufficient light. This is because poor lighting makes workers move into awkward positions to see what they are doing. This condition makes workers especially that involve in sewing machines, spinning and weaving sections who demands visual concentration to increase the risk of developing MSDs.

### Conclusions

This study indicates that the prevalence of back musculoskeletal disorders among production workers was high. Gender, age, years of service, lack of physical activity, unavailability of adjustable chair, work load and poor light were significantly associated with increased risk of back pain. The high prevalence of work-related back pain disorder implies that; habit of doing physical exercise, availing adjustable chair and light at the working place, are key issues which require specific interventions.

## Limitations

This study encountered some limitations; lack of using measurement tool for variables like: light intensity. Since the 1 year prevalence of MSDs is based on self reporting of workers, there could be problem of recall bias and may underestimate the results.

## Additional files

**Additional file 1.** Questionnaire English version. The questionnaire uploaded as Additional file 1 used to assess predictors of back disorder among Almeda textile factory workers, North Ethiopia.

**Additional file 2.** Predictors of back disorder SPSS data.

## Abbreviations

CI: confidence interval
LBP: lower back pain
MSDs: musculo skeletal disorders
PLC: Private Limited Company
WHO: World Health Organization
