# Supplementary material for: Predictors of back disorder among Almeda textile factory workers, North Ethiopia
Source: BMC Res Notes. 2018 May 16;11:304. doi: 10.1186/s13104-018-3440-4 (PMC5956613; doi:10.1186/s13104-018-3440-4)
Supplement: Supplementary file 1 — Additional file 1. Questionnaire English version. The questionnaire uploaded as Additional file 1 used to assess predictors of back disorder among Almeda textile factory workers, North Ethiopia. [file 13104_2018_3440_MOESM1_ESM.docx]

## Annex 1. English version verbal consent form

**Written consent form**

How are you, my name is ………………………………….. I am here on behalf of Teklehaymanot Huluf Abraha, he is conducting a research for the partial fulfillment for the on Predictors of back disorder among Almeda textile factory workers, North Ethiopia. He has received permission from Institute of public health at university of Gondar to conduct this study. The main part of the study involves collecting information about Predictors of back disorder among Almeda textile factory workers, North Ethiopia like you. You were selected for the study because you are worker of Almeda Textile Factory with the hope that you will cooperate with us. Your status on back, shoulder and neck disorder and your practices in your work will help to solve the problem of back, shoulder and neck disorders and improve the work environment. We are kindly requesting you to answer the questions that we have prepared for you. Your name will not be written in this form and will never be used in connection with any information you tell us. All information given by you will be kept strictly confidential. Your participation is voluntary and you are not obligate to answer any question you do not wish to answer. If you fill discomfort with the interview, please fill free to drop out any time you want. This face to face interview questionnaire will take around 20 minutes. Could I have your permission to continue?

1. If yes, continue the interview.

2. If no, skip to the next participant by writing the reasons for his/her refusal.

Informed consent Certified by:

Interviewer: Code-----------------Name-----------------------------signature---------------

Date of interview-----------------Time started--------------------Time completed-------

Result of interview:

1. Completed 2.Respondent not available, 3, Refused, 4. Partially completed

Checked by:

Supervisor Name--------------------------signature----------------Date------

Questionnaire identification number --------------

## Annex 2. Information Sheet and Consent Form:

This information sheet is prepared to explain the research project that you are asked to join by a group of investigators. The main aim of this research project is to assess the Predictors of back disorder among Almeda textile factory workers, North Ethiopia**.**

**Name of Principal investigator:** Teklehaymanot Huluf Abraha (MPH)

**Name of the organization:** University of Gondar, College of Medicine and Health Sciences, Institute of Public Health**.**

**Co Authors:** Mr. Asmelash Tekie

Mr. Haimanot Gebrehiwot (Ass.Prof)

Mrs. Ansha Nega (Ass.Prof**)**

**Name of the Sponsor:** University of Gondar

**Introduction**: These information sheet and consent forms are prepared to explain the study you are being asked to join. Please listen carefully and ask any questions about the study before you agree to join. You may ask questions at any time after joining the study.

**Purpose of Research Project**: The purpose of this research is to assess the Predictors of back disorder among Almeda textile factory workers, North Ethiopia. The study will be helpful in determining the magnitude of work-related back disorder WMSD and identifying the associated factors and contribute to design appropriate intervention strategies related to back, shoulder and neck WMSDs.

**Procedure**: To assess back of WMSD, we invite you to take part in this project. If you are willing to participate in this project, you need to understand and sign the agreement form. Then after, you will be interviewed by the data collector to give your response. You do not need to tell your name to the data collector and all your responses and the results obtained will be kept confidentially by using a coding system whereby no one will have access to your response.

**Risk/ Discomfort**: By participating in this research project, you may feel that it has some discomfort especially on wasting time about 30 minutes. We hope you will participate in the study for the sake of the benefit of the research result. There is no risk in participating in this research project.

**Benefits**: If you participate in this research project, there may not be direct benefit to you but your participation is likely to help us in assessing the back, shoulder and neck WMSDs.

**Incentives**: You will not be provided any incentives or payment to take part in this project.

**Confidentiality**: The information collected from this research project will be kept confidential and information about you that will be collected by this study will be stored in a file, without your name, but a code number assigned to it. And it will not be revealed to anyone except the principal investigator and will be kept locked with key.

**Right to refuse or withdraw**: You have full right to refuse from participating in this research. You can choose not to respond to some or all questions if you do not want to give your response. You have also the full right to withdraw from this study at any time you wish, without losing any of your rights.

**Person to contact:** This research project will be reviewed and approved by the ethical committee of the University of Gondar. If you have any question you can contact the following individuals and you may ask at any time.

Teklehaymanot Huluf Abraha (MPH)

<Tel:+251912890608>

Email: teklehaymanothuluf@gmail.com

Asmelash Tekie

Tel: +251-942043424

Email: [saynesasie@gmail.com](mailto:saynesasie@gmail.com)

Mr. Haimanot Gebrehiwot

Tel: +251-910580545

Email: haminot_ghiwot@yahoo.com

Ms. Ansha Nega

Tel: +251-918151073

Email: [anshanega@yahoo.com](mailto:anshanega@yahoo.com)

## Annex 3. English version questionnaire

**Part I.** Questions on socio-demographic characteristics.

| S/ No | Questions | Choice Answers |
| --- | --- | --- |
| 101 | Department | 1. Spinning 2. Weaving 3. Garment 4. Dyeing |
| 102 | Sex | 1. Male 2. Female |
| 103 | Age | -----------years |
| 104 | Marital status | 1. Married 2. Single 3. Divorced 4. Separated 5. Widowed |
| 105 | Educational status | 1. Illiterate 2. Primary school 3. Secondary School 4. Technical/vocational 5. Higher Education |
| 106 | Monthly Salary | --------------in birr |
| 107 | Work Experience in textile industry | _________in years |

**Part II**. Questions for Organizational factors

| S/No | Question | Answers |
| --- | --- | --- |
| 201 | Employment status | 1. Temporary 2. Permanent |
| 202 | Total working hours a day | 1. ≤ 8 hours 2. 9-10 hours 3. >10 hours |
| 203 | Total working break (excluding lunch break) per day | 1.≤ 15 minute  2. > 15minute  3.None |
| 204 | Have you ever attended occupational health and safety training on ergonomics? | 1. Yes 2. No |

**Part III.** Questions on Personal factors

| S/No | Question | | Answer |  |
| --- | --- | --- | --- | --- |
| 301 | Height | | -------------cm |  |
| 302 | Weight | | -------------K.g |  |
| 303 | How many times you are doing physical activity (none work) per week? | | 1. None 2. Once per week 3. three times per week 4. ≥ 4 times per week |  |
| 304 | Do/did you smoke? | | 1. None 2. Occasionally 3. 1-3 days/week 4. Every day |  |
| 305 | Does your task demand lifting? | | 1. Yes 2. No | If no  go to Q307 |
| 306 | Do you have safe lifting mechanism? | | 1. Yes 2. No |  |
| 307 | Does your task demand prolonged sitting?  If your answer is ‘No’ go to Q209 | | 1. Yes 2. No | If no  go to Q309 |
| 308 | Do you have the habit of proper sitting? | | 1. Yes 2. No |  |
| 309 | Does your task demand prolonged standing? | | 1. Yes 2. No | If no  go to Q311 |
| 310 | Do you have the habit of proper standing? | | 1. Yes 2. No |  |
| 311 | Do you Have the history of MSDs before employment? | Back | 1. Yes 2. No |  |
|  |  | Shoulder | 1. Yes 2. No |  |
|  |  | Neck | 1. Yes 2. No |  |
| 312 | Is there any heredity of MSDs in your family? | Back | 1. Yes 2. No |  |
|  |  | Shoulder | 1. Yes 2. No |  |
|  |  | Neck | 1. Yes 2. No |  |

**Part IV.** Questions related to back, shoulder and neck musculoskeletal symptoms

| S/No | Question | | Answer |  | |
| --- | --- | --- | --- | --- | --- |
| 401 | Have you at any time during the last 12 months had trouble ache, pain, and discomfort on your Back? | | 1. Yes 2. No | If no  go to Q405 | |
| 402 | Have you at any time during the last 12 months hurt your back in accident? | | 1. Yes 2. No |  | |
| 403 | Which part of your back had trouble, ache, pain, and discomfort? | | 1. Lower back 2. Upper back |  | |
| 404 | What is the total length of time that you have had back trouble during the last 12 months?   | Lower back | 1. 0 days 2. 1-7 days 3. 8-30 days 4. >30 days, but not every day 5. Every day | |  |
|  |  | Upper back | 1. 0 days 2. 1-7 days 3. 8-30 days 4. >30 days, but not every day 5. Every day | |  |
|  |  | both | 1. 0 days 2. 1-7 days 3. 8-30 days 4. >30 days, but not every day 5. Every day | |  |
| 405 | Have you at any time during the last 12 months had trouble ache, pain, and discomfort on your Shoulder? | | 1. Yes 2. No | | If no  go to Q408 |
| 406 | Have you at any time during the last 12 months hurt your shoulder in accident? | | 1. No 2. Yes | |  |
| 407 | What is the total length of time that you have had shoulder trouble, ache, pain, and discomfort during the last 12 months? | | 1. 0 days 2. 1-7 days 3. 8-30 days 4. >30 days, but not every day 5. Every day | |  |
| 408 | Have you at any time during the last 12 months had trouble ache, pain, and discomfort on your Neck? | | 1. Yes 2. No | | If no  go to Q501 |
| 409 | Have you at any time during the last 12 months hurt your neck in accident? | | 1. No 2. Yes | |  |
| 410 | What is the total length of time that you have had neck trouble ache, pain, and discomfort during the last 12 months? | | 1. 0 days 2. 1-7 days 3. 8-30 days 4. >30 days, but not every day 5. Every day | |  |

**Part V.** Questions for Working Environment/Condition and ergonomic factors

| S/No | Question | | Answer |  |
| --- | --- | --- | --- | --- |
| 501 | Does your task demand repetitive work? | | 1. Yes 2. No | If no  go to Q503 |
| 502 | How much is its repetition? | | 1. < 30 seconds 2. > 30 seconds |  |
| 503 | Do you have work load? | | 1. Never 2. Sometimes 3. Always |  |
| 504 | Are you satisfied with your current job? | | 1. Not satisfied 2. Somewhat satisfied 3. Very satisfied |  |
| 505 | Does Your work demand visual concentration? | | 1. Yes 2. No | If no  go to Q507 |
| 506 | How much lighting is available? | | 1. Low(almost no need to see fine details, >50cm 2. High( need to see fine details,<50cm) |  |
| 507 | Does your task involve weight to handle? | | 1. Yes 2. No | If no  go to Q510 |
| 508 | What is the maximum weight handled in this task? | | 1. Light(5 kg or less) 2. Moderate (6-10 kg) 3. Heavy (11-20 kg) |  |
| 509 | How much time on average do you spend per day doing this task? | | 1. < 2 hours 2. 2 to 4 hours 3. > 4 hours |  |
| 510 | Is your chair an adjustable? | | 1. Yes 2. No |  |
| 511 | For how many hours per day/shift do you sit in performing your task? | | 1. None 2. < 6 hours(low) 3. > 6 hours(high) |  |
| 512 | For how many hours per day/shift do you stand in one location in performing your task? | | 1. None 2. < 4 hours(low) 3. > 4 hours(high) |  |
| 513 | Does your work involve | Frequent bending or twisting | 1. Yes 2. No |  |
|  |  | Reaching over shoulder | 1. Yes 2. No |  |
|  |  |  |  |  |

Thank you very much.
